# The expression and roles of Nde1 and Ndel1 in the adult mammalian central nervous system

**DOI:** 10.1016/j.neuroscience.2014.04.031

**Published:** 2014-06-20

**Authors:** Z. Pei, B. Lang, Y.D. Fragoso, K.D. Shearer, L. Zhao, P.J.A. Mccaffery, S. Shen, Y.Q. Ding, C.D. McCaig, J.M. Collinson

**Affiliations:** a School of Medical Sciences, Institute of Medical Sciences, University of Aberdeen, Foresterhill, Aberdeen AB25 2ZD, United Kingdom; b Department of Neurology, Medical Faculty, Universidade Metropolitana de Santos, Sao Paulo, Brazil; c Regenerative Medicine Institute, School of Medicine, NUI Galway, Galway, Ireland; d Tongji University School of Medicine, 1239 Siping Road, Shanghai 200092, China

**Keywords:** BSA, bovine serum albumin, CNV, copy number variations, Disc1, disrupted in schizophrenia 1, FBS, fetal bovine serum, Nde1, nuclear distribution factor E, Ndel1, nuclear distribution protein nudE-like 1, PBS, phosphate-buffered saline, RT-PCR, reverse-transcription polymerase chain reaction, SGZ, subgranular zone, SVZ, subventricular zone, expression, Nde1, Ndel1, adult neurogenesis, HCNA94, differentiation

## Abstract

Mental and neurological illnesses affect one in four people. While genetic linkage analyses have shown an association of *nuclear distribution factor E* (*NDE1*, or *NudE*) and its ohnolog *NDE-like 1* (*NDEL1*, or *Nudel*) with mental disorders, the cellular mechanisms remain unclear. In the present study, we have demonstrated that Nde1 and Ndel1 are differentially localised in the subventricular zone (SVZ) of the forebrain and the subgranular zone (SGZ) of the hippocampus, two regions where neurogenesis actively occurs in the adult brain. Nde1, but not Ndel1, is localized to putative SVZ stem cells, and to actively dividing progenitors of the SGZ. The influence of these proteins on neural stem cell differentiation was investigated by overexpression in a hippocampal neural stem cell line, HCN-A94. Increasing Nde1 expression in this neural stem cell line led to increased neuronal differentiation while decreasing levels of astroglial differentiation. In primary cultured neurons and astrocytes, Nde1 and Ndel1 were found to have different but comparable subcellular localizations. In addition, we have shown for the first time that Nde1 is heterogeneously distributed in cortical astrocytes of human brains. Our data indicate that Nde1 and Ndel1 have distinct but overlapping distribution patterns in mouse brain and cultured nerve cells. They may function differently and therefore their dosage changes may contribute to some aspects of mental disorders.

## Highlights

• This is the most detailed morphological documentation of Nde1 and Ndel1 both *in vivo* and *in vitro*.
• This is the first histological study of Nde1 expression in the healthy human cortex.
• We demonstrate the localization of Nde1 and Ndel1 in neurogenic regions of the adult mouse brain.
• Overexpression of Nde1 and Ndel1 leads to the fate specification changes of neural stem cells.

## Graphical abstract

Nde1 (red) is localised to the GFAP-positive (green) stem cell of the subventricular zone of the lateral ventricle, and its overexpression in neural stem cells promotes neuronal differentiation while inhibiting astroglial differentiation.

**Figure:**
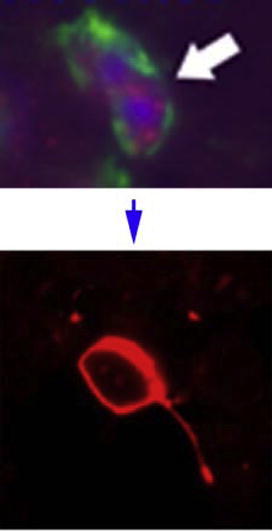

## Introduction

Nuclear distribution factor E (Nde1, or NudE) and nuclear distribution factor E-like (Ndel1, or Nudel) are ohnologous, evolutionarily conserved proteins with more than 50% amino acid conservation (Sweeney et al., 2001). They are coiled-coil proteins that can form a complex by binding lissencephaly-1 (LIS1) (Derewenda et al., 2007), and can also interact with dynein. They are essential for the function of microtubules which regulate cell mitosis and migration (McKenney et al., 2010). Nde1 and Ndel1 are both critical for cortical development. They regulate developmental neurogenesis and cortical neuronal positioning (Shu et al., 2004; Tsai et al., 2005, 2007). For example, *NDE1* mutations in humans can cause lissencephaly, a cortical developmental disease, and overt microcephaly (Alkuraya et al., 2011; Bakircioglu et al., 2011). Targeted deletion of *Nde1* in mice is often embryonically or postnatally lethal and the mice have fewer cortical neurons and a much thinner cortex, reminiscent of lissencephaly. Mutation in *Nde1* reduces the neural progenitor pool, dramatically inhibits later stage neuronal migration and blocks the development of the upper cortical layers (Feng et al., 2000; Feng and Walsh, 2004). *Ndel1-*null mutant mice are also embryonic lethal – *Ndel1*^−/−^ embryos die shortly after implantation. *Ndel1* null/hypomorph compound heterozygotes (*Ndel1^cko/−^*) exhibit mild neuronal migration defects (Shu et al., 2004; Sasaki et al., 2005). In addition, Ndel1 can be phosphorylated by Aurora A kinase at position S251 or by the Cdk1/Cdk5 kinase at positions T219/T245, which are important for the interaction with dynein (Zylkiewicz et al., 2011) and for the regulation of neuronal migration (Mori et al., 2007).

The investigation of copy number variation (CNV) of *NDE1* and *NDEL1* strongly suggests that they are associated with neuropsychiatric disorders. In genetic association studies, *NDE1* has been identified as a strong candidate for epilepsy (Heinzen et al., 2010) and schizophrenia (Ekholm et al., 2003; Hennah et al., 2007; Ingason et al., 2011). The Disrupted in schizophrenia 1 (DISC1) protein is a common binding partner of NDE1 and NDEL1. Both *NDEL1* and *DISC1* are important genetic risk factors for the development of schizophrenia (Ozeki et al., 2003; Burdick et al., 2008), and they are all involved in regulating the migration of newly generated neurons in the adult hippocampus. Downregulation of *DISC1* in stem cells in the hippocampal subgranular zone (SGZ) led to the formation of ectopic primary dendrites and mispositioning of the soma during neuronal maturation (Duan et al., 2007). Although Nde1 is involved in epilepsy, and adult hippocampal neurogenesis is up-regulated in rodent epilepsy models (Bengzon et al., 1997; Parent et al., 1997; Jessberger et al., 2007) and after antidepressant treatment (Malberg et al., 2000; Reif et al., 2006), the roles of Nde1 in these processes are less intensively investigated and therefore remains unclear.

Intriguingly, although there are several sporadic reports in the embryonic mouse and human cortex (Bakircioglu et al., 2011; Pawlisz and Feng, 2011), little information for the localization of Nde1 and Ndel1 in the developing and adult brains is available. It is essential to perform more wide and detailed investigations of the expression pattern of Nde1 and Ndel1 in the adult brain. For instance, its expression and dysfunction in astrocytes might play important roles in neurological disorders including epilepsy (Blumcke et al., 2002; de Lanerolle and Lee, 2005; Seifert and Steinhauser, 2013) and schizophrenia (Rothermundt et al., 2004). Although *NDE1* and *NDEL1* have been shown to be expressed in some human gliomas, their expression in normal astrocytes has not been well described.

In the present study, we have performed a comprehensive regional comparison of the expression profiles of Nde1 and Ndel1 in the adult mouse brain, with emphasis on the subventricular zone (SVZ) in the forebrain and the SGZ in the hippocampus, two regions where adult neural stem cells reside. We also analyzed the distribution and function of Nde1 and Ndel1 in primary neuronal/glia culture and a hippocampal neural stem cell line, HCN-A94. Our further functional studies suggest that overexpression of Nde1 and Ndel1 play important roles in cell fate specification, which potentially links with a range of neurodevelopmental and neuropsychiatric disorders.

## Experimental procedures

### Animals and section preparation

All the animal work was approved by the Local Ethics Review Board of the University of Aberdeen. All animal procedures were performed with UK Home Office licence under the Animals (Scientific Procedures) Act 1986, with guidance of the Medical Research Facility, Aberdeen University, UK. Particular care was taken to minimize the number of animals to achieve statistical significance. Human brain tissue work in the present study was approved by the Ethics Committee of Universidade Metropolitana de Santos, SP, Brazil and by the Brazilian Health Research Committee on 4th of April 2011, under the number CONEP 16168, documents registered under the number 25000.169694/2010-18. Human brain tissue samples were collected from prefrontal, frontal and temporal cortices of adult male individuals aged 55 years or less at the Guilherme Alvaro Hospital, Santos, SP, Brazil, with written consent from the relatives of the deceased. None of the subjects presented a major neurological or psychiatric condition during life and the cause of death excluded head trauma, brain infection and stroke.

For immunohistochemistry, the brains of adult C57BL/6J mice were dissected out, embedded in Optimal Cutting Temperature compound (OCT) and snap-frozen on dry ice (*n* = 3). Serial coronal sections with thickness of 12 μm were yielded in a cryostat (Leica, CM1850, Germany) and mounted on the poly-lysine-coated slices (VWR, UK). These sections were stored in −80 °C just before use.

### Immunohistochemistry

Mouse brain sections were post-fixed in 4% paraformaldehyde (pH 7.4) for 5 min and washed with phosphate-buffered saline (PBS). Antigen retrieval was carried out by incubation in 0.01 M sodium citrate buffer (pH 6.0) and microwaving (800 W, 6 min). Non-specific immunoreactivity was suppressed by incubating samples in 5% bovine serum albumin (BSA) (Sigma, UK) and 5% serum of the species which generated the secondary antibody. For immunofluorescent staining, the following primary antibodies were used: mouse anti-GAD67 (1:1000; Millipore, US), mouse anti-GFAP (1:1000; Abcam, UK), rabbit anti-GFAP (1:2000; DAKO, Denmark), rat anti-Ki67 (1:100; DAKO, Denmark), mouse anti-MAP2 (1:3000; Sigma, UK), rabbit anti-MAP2 (1:200; Cell Signalling, US), rabbit anti-Nudel (1:400; Abcam, UK), rabbit anti-Nde1 (1:400; Sigma, UK), goat anti-Ndel1 (1:400; Sigma, UK), mouse anti-Nestin (1:200; Abcam, UK), mouse anti-NeuN (1:400; Millipore, US), goat anti-SOX2 (1:200; Santa Cruz, US), mouse anti-Vimentin (1:100; DSHB, US), rabbit anti-β tubulin III (1:3000; Sigma, UK) and mouse anti-β tubulin III (1:3000; Sigma, UK). The primary antibodies were diluted in PBS with 3% BSA plus 0.1% Triton X-100 and applied for 24 h at 4 °C. Sections were then incubated with different combinations of fluorescent-conjugated secondary antibodies (Molecular Probes, US), which included Alexa 488-conjugated donkey anti-goat, donkey anti-rabbit, donkey anti-mouse and goat anti-mouse IgG; Alexa 594-conjugated donkey anti-goat, donkey anti-mouse, donkey anti-rabbit and donkey anti-rat IgG; and Cy3-conjugated donkey anti mouse IgM. The sections were finally counterstained with Hoechst 33258 (Molecular Probes, US).

To visualize the immunoreactivity, some sections were incubated with biotin-conjugated secondary antibodies (Sigma, UK) followed by Vectastain Elite avidin/biotin complex reagent (Vector Laboratories, UK) according to the manufacturer’s instructions. After being washed with PBS, immunoreaction was visualized with 3,3′-diaminobenzidine (Sigma, UK). One of each series of the immunostained sections was counterstained with cresyl violet for the study of cytoarchitecture. Internal standards were employed to evaluate the intensity of immunostaining semi-quantitatively by two independent researchers without the knowledge of the experiment. The intensity of immunoreactivity was categorized as intense (+++), moderate (++), weak (+) and negative (−), which represented the relative expression levels among different regions and had no indication of the actual amount of the protein. The negative staining was performed with the same protocol except that the primary antibodies were replaced with normal IgG from the same animal species. The anatomical nomenclature of Paxinos and Watson (1986) was adopted to describe the regions of the CNS.

Immunofluorescence staining was also performed on 7-μm thick human prefrontal, frontal and temporal sections prepared from formalin-fixed and paraffin-embedded blocks. Sections were de-waxed in histoclear (National Diagnostics, US) and rehydrated through decreasing ethanol concentrations (100%, 95%, 80% and 70%). Antigen retrieval was carried out by incubating sections in sodium citrate buffer (pH 6.0) and microwaving (800 W, 6 min) followed by incubation in ice-cold NaHBO_4_ (0.5%, in 0.1 M pH 7.4 phosphate buffer, 10 min). Blocking and subsequent steps followed the same protocol as for mouse tissue fluorescence staining. Lipofuscin autofluorescence was quenched by applying Sudan Black-B (1% in 70% ethanol). The samples were then mounted in Aquamount (DAKO, Denmark).

### Western blotting

Adult mouse brains were dissected into seven parts: olfactory bulb (OB), cortex (Ctx), thalamus (Tha), hippocampus (Hp), cerebellum (Cereb, Cb), pons and spinal cord (Sp). Tissue samples were lysed in cell lysis buffer (Cell Signaling, US) containing a cocktail of protease inhibitors (Complete™, Roche, UK). The lysates were analyzed by 14% sodium dodecyl sulfate polyacrylamide gel electrophoresis (SDS–PAGE) (15 μg protein/sample). Membranes were blocked in skimmed milk for 1 h and exposed to the primary antibodies overnight at 4 °C. The primary antibodies are rabbit anti-Nudel (1:1000; Abcam, UK), rabbit anti-Nde1 (1:1000; Sigma, UK), goat anti-Ndel1 (1:1000; Sigma, UK). Mouse anti-GAPDH (1:10,000, Santa Cruz, US) or mouse anti-α-tubulin (1:50,000; Sigma, UK) were used as loading controls. After secondary antibody incubation (1 h), the membranes were developed by an enhanced chemiluminescence kit (Millipore, US).

### *In vitro* over-expression of Nde1/Ndel1

Rat Nde1 or Ndel1 coding sequences (CDS) were reverse-transcription polymerase chain reaction (RT-PCR)-amplified from mRNA and inserted independently into the pEGFP-C1 vector (Clontech, US) between the BspEI and SalI restriction sites. For Nde1, the following primers were used: forward (BspEI site), 5′-GCC-TCCGGA-ATGGAGGACTCGGGAAAGACTT-3′; reverse (include SalI site), 5′-GGT-GTCGAC-GCAGTTCCCACTGGTCCTAAAGC-3′. For Ndel1, forward (include SalI site), 5′-TTC-GTCGAC-ATGGATGGTGAAGATATACCGGAT-3′; reverse (include BamHI site), 5′-ACC-GGATCC-CCGAGGGACGAGGCGTA-3′. Untreated pEGFP-C1 vectors were prepared for negative control. All the constructs were sequenced to confirm integrity.

The HCN-A94 cell line was a gift from Prof. Fred H. Gage (Salk Institute, US) and was maintained as described (Gage et al., 1995; Shen et al., 2011). DMEM medium with 0.5% fetal bovine serum (FBS), 1 μM transretinoic acid and N-2 (Invitrogen, US) were used for differentiation. To achieve *in vitro* overexpression, undifferentiated HCN-A94 cells were transfected with the pCMV-Nde1-EGFP, pCMV-Ndel1-EGFP or empty pCMV-EGFP vectors by using the rat neural stem cell Nucleofector kit (Lonza, UK). From the second day after transfection, the expression of constructs was maintained by applying selective antibiotic G418 (Sigma, 400 μg/ml) into the culture. The same dose of G418 also was used during the process of differentiation.

### Primary culture and immunocytochemistry

Mouse (C57BL/6) cortices and hippocampi were dissected at the embryonic stage E18.5. Neuronal and glial primary culture was established as described previously (Viesselmann et al., 2011). Primary neuronal cells were maintained in Neurobasal-A medium (Invitrogen) supplemented with GlutaMax (Invitrogen, US) and B27 (Invitrogen, US) up to the time points of analysis (24 h, day 2, day 4, day 7). Primary astrocytes were plated on poly-l-lysine-coated glass coverslips (13 mm, VWR, UK) at a density of 3000 cells per ml and maintained in DMEM-F12 medium with 10% FBS. They were cultured for two weeks before analysis.

Both the HCN-A94 cell lines and the primary cultured neurons/astrocytes were fixed in paraformaldehyde (4%) for 5 min, permeabilized in −20 °C methanol for 2–3 min and treated with blocking buffer (5% donkey serum, 0.3% Triton X-100 in PBS) at room temperature for 1 h. Immunofluorescence and negative staining were performed using the same protocol as mouse tissue staining.

### mRNA analysis by RT-PCR

RNA of undifferentiated HCN-A94 cells was isolated with the Total RNA Kit (Peqlab, UK) and was DNaseI- (Roche, UK) digested according to the manufacturers’ instructions. cDNA was synthesised by using SuperScript II (Invitrogen, US) and oligo dT12–18 primer (Amersham Biosciences, UK). Amplification was performed with 1 ng cDNA or the same volume control samples. The following primers were used: for Nde1, forward: 5′-TGGCCCAGTCCCTAGCAGTGG-3′; reverse: 5′-GGCCAGTCCAACGCAAGCTGT-3′; for Ndel1, forward: 5′-GGCGGCGAACATGCGCTTTT-3′; reverse, 5′-TGTTGCCCGTTTTGCTCGCT-3′. The annealing temperature was 60 °C.

### Quantitative-RT PCR analysis of human cortical samples

Samples of human prefrontal, frontal and temporal cortex were collected in RNAlater RNA Stabilization Reagent (Qiagen, Venlo, The Netherlands) and stored at 4 °C for qPCR. RNA extraction and quantitative PCR (qPCR) analysis followed our previously published protocols (Fragoso et al., 2012). The following primers were used. For NDE1: forward, 3′-AGCCAGAGATTTGCGGCAGGAAC-5′; reverse: 3′-CCTCGGTGAGCAATGGGCGT-5′. For NDEL1: forward, 3′-GGGATCTCTTACGGAAAGTAGGGGCTT-5′; reverse, 3′-GCCATTGCCATTCAGCACCCC-5′. The annealing temperature was 60 °C.

### Image analysis

To quantify neural and astrocytic differentiation ratio in over-expression experiments, fifteen microscopic fields (10× objective) were picked randomly in each sample and the number of GFP single-labeled cells, GFAP/GFP double-labeled cells or Tuj1/GFP double-labeled cells were counted in each field. The proportion of differentiated astrocytes with GFP was calculated by the number of GFAP/GFP double-labeled cells against the total number of GFP single-labeled cells. The proportion of neuronal differentiation was presented by the ratio of the number of Tuj1/GFP double-labeled cells against the total number of GFP single-labeled cells. The number of total GFP-positive cells is larger than 10,000 in each group. The experiment was repeated three times. Results of Nde1-overexpressing group or Ndel1-overexpressing group were compared independently with the pCMV-EGFP vector-transfected group (negative control).

To quantify the NDE1 expression ratio in astrocytes of white matter or gray matter in the human cortex, two different cortical sections were taken from each donor. Ten to fifteen microscopy fields (10× objective) were taken randomly in gray matter or white matter and the number of NDE/GFAP double-labeled and GFAP single-labeled cells was counted. For each section, the number of NDE/GFAP double-labeled cells was divided by the number of GFAP single-labeled cells. The results were then analyzed between gray matter and white matter by the Mann–Whitney U test. *p* < 0.05 was considered as significantly different.

## Results

### Expression of Nde1 and Ndel1 in the adult mouse CNS

**Fig. 1:**
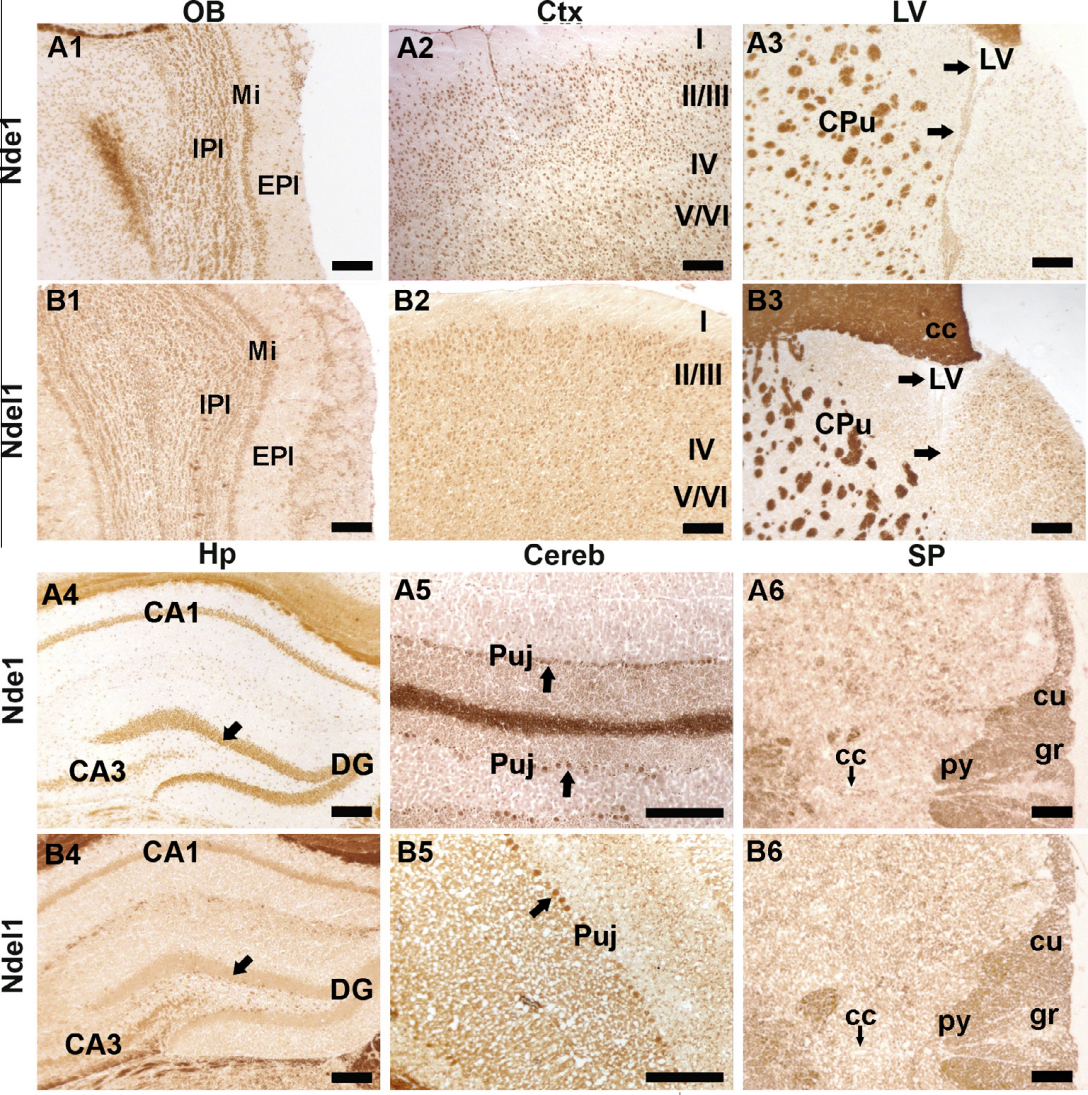
Distribution of Nde1 and Ndel1 in the adult mouse CNS. (A–B) Immunohistochemical staining shows that Nde1 (A1–A6) and Ndel1 (B1–B6) are distributed widely in adult mouse CNS. Arrows in A3 and B3 indicate the lateral ventricle area. Arrows in A4, B4 show the granular layer of dentate gyrus. Arrows in A5 and B5 show the Purkinje cell layer. Arrows in A5 and B5 indicate the central canals in the cervical spinal cord. Strongest staining was also found in projecting nerves and the major white matter tracts. Negative controls showed only faint background staining after overdevelopment. I–VI, sublayers of cortex; cc in B3, corpus callosum; cc in A6 and B6, central canal; Cereb, cerebellum; CPu, caudate putamen; Ctx, cortex; cu, cuneate fasciculus; DG, dentate gyrus; MI, mitral cell layer; EPI, external plexiform layer; gr, gracile fasciculus; Hp, hippocampus; IPI, internal plexiform layer; LV, lateral Ventricle; OB, olfactory bulb; Puj, purkinje cell layer; py, pyramidal tract; SP, spinal cord. Scale bar = 100 μm.

**Fig. 2:**
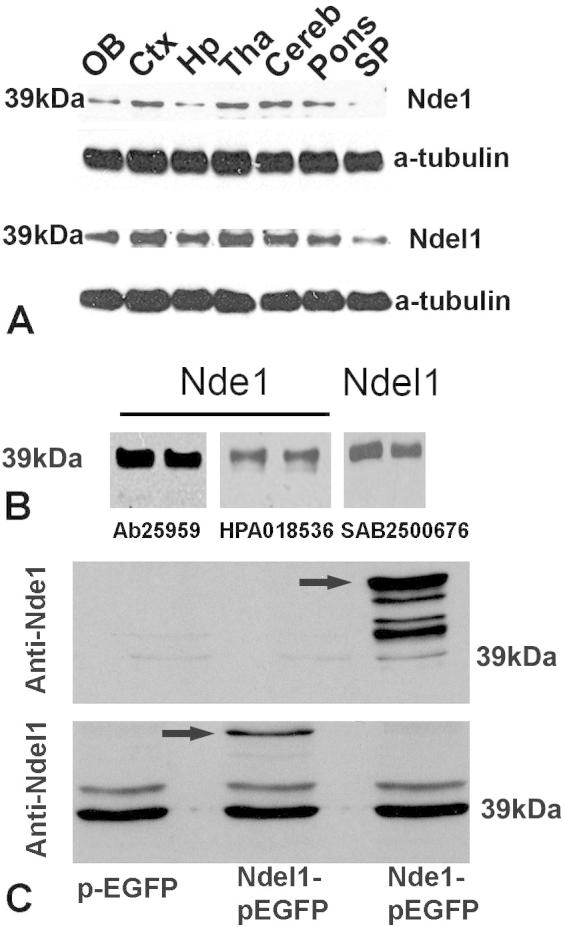
Western blot analysis of Nde1 and Ndel1 profiles. (A) Immunoblotting was carried out on lysates from different sub-regions of the adult mouse brain and robust expression of Nde1 (39 kDa) and Ndel1 (39 kDa) were detected. (B) Lysates of the adult mouse cortex (two independent lanes for each antibody) were blotted with anti-Nde1 (Ab25959, Abcam, left lane), anti-Nde1 (HPA018536, Sigma, middle lane) and anti-Ndel1 (SAB2500676, Sigma, right lane). These antibodies all detected one band of the same size which highlighted their unique specificities. (C) Cell lysates from NIH3T3 cells transiently transfected with pCMV-EGFP (left lane) or with pCMV-Ndel1-EGFP (middle lane) or with pCMV-Nde1-EGFP (right lane) were subject to the blotting with anti-Nde1 (HPA018536, top) or anti-Ndel1 (SAB2500676, bottom). Ndel1 antibody detected abundant Ndel1 expression in 3T3 cells, whereas Nde1 antibody showed little expression of Nde1 in 3T3 cells. Little or no cross-reaction between anti-Nde1 and Ndel1 further confirmed the specificity of these two antibodies. Several extra bands were also detected underneath Nde1-EGFP fusion protein in top panel of C. They were likely the degraded products of the fusion protein during protein preparation. Arrows indicate EGFP fusion proteins.

Although Nde1 and Ndel1 play important roles in neurodevelopment and neuropsychiatry, little information is available about their expression and distribution in the adult brain. We therefore performed immunohistochemical studies and investigated the detailed localization of Nde1 and Ndel1 in adult mouse brains. Nde1 and Ndel1 were found widely distributed in the CNS of adult mice. Their distribution patterns were similar but not identical (Fig. 1). Immunodouble-labeling showed that both proteins were largely co-expressed with the neuronal marker NeuN in CNS (Fig. 4C, D, I). In the olfactory bulb, Nde1 and Ndel1 were strongly expressed within the internal/external plexiform layers, mitral cell layer and granular cell layer (Fig. 1A1, B1). They were also detected in most cortical neuronal cells (Fig. 1A2, B2), the lateral ventricle regions (especially for Nde1, Fig. 1A3) and in the hippocampus (Fig. 1A4, B4). Nde1 and Ndel1 proteins were not detected to the same intensity in all brain regions. For example, in the hippocampus, Nde1 was present at high levels in CA1–3, the dentate gyrus granular cell layer (arrowed) and the polymorph layer. In contrast, though Ndel1 staining revealed a slightly higher expression in the polymorph layer, Ndel1 is only moderately expressed in CA1–3 and dentate gyrus (Fig. 1A4, B4, arrowed). In the cerebellum, somatic expression was most abundant in Purkinje neurons (Fig. 1A5, B5, arrows). Both proteins were broadly and strongly localized in white matter tracts, and projecting nerves, including in the spinal cord (Fig. 1A6, B6). We further performed western blotting assays and also detected robust expression of Nde1 and Ndel1 in different brain sub-regions (Fig. 2). Two antibodies against different peptide sequences of Nde1 were used and the results were consistent. A detailed summary of the distribution of Nde1 and Ndel1 proteins in the mouse CNS is given in Table 1.

### Distribution of Nde1 and Ndel1 in the adult mouse SVZ

**Fig. 3:**
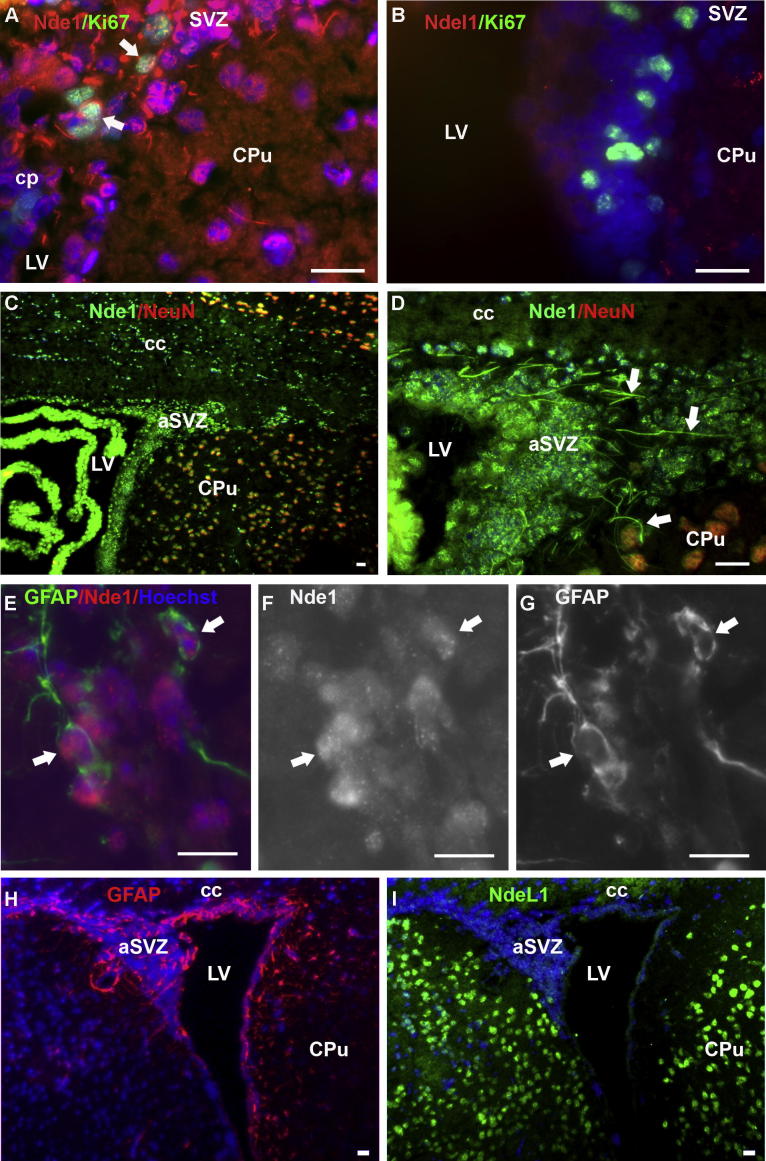
Distribution profiles of Nde1 and Ndel1 in the adult SVZ. (A, B) Immunohistochemistry of adult mouse brain slices showed that Nde1 but not Ndel1 is distributed widely in the SVZ. Nde1 also is expressed weakly in Ki67-positive cells (white arrows). (C, D) Nde1-positive cells in the SVZ are not NeuN-expressing mature neurons. Some of these cells have long processes which contained Nde1 (white arrows). (E–G) Subsets of Nde1-positive cells in SVZ also express GFAP (white arrows). (H, I) Ndel1 is not highly expressed in the SVZ and is not co-expressed with GFAP in this region. cc, corpus callosum; cp, choroid plexus; CPu, caudate putamen; LV, lateral ventricle; LSV, lateral septal nucleus, ventral; aSVZ, anterior subventricular zone; Scale bar = 20 μm in A–D, H, and I; 25 μm in E–G.

Because Nde1 functions in stem cell mitosis (Feng and Walsh, 2004), we hypothesized that Nde1 may be expressed in the adult stem cells of SVZ *in vivo*. Two subtypes of SVZ neural progenitors have been identified so far: (1) infrequently or slowly dividing type B cells (putative stem cells) which are GFAP-positive and share some characteristics with radial glia; (2) type C active mitotic cells (Suh et al., 2009) which express Ki67 instead of GFAP. Our results showed that Nde1 (not Ndel1) was extensively expressed in the adult mouse SVZ (Fig. 3), and weakly co-localized with Ki67, a proliferation marker for actively dividing cells in the SVZ (Fig. 3). There was no co-localization of Nde1 and NeuN, markers for mature neurons, in the SVZ (Fig. 3C, D). In addition, some of the Nde1-stained cells had a unique radial process (Fig. 3D, white arrows) which may indicate astrocytic or stem cell morphology. We speculated that the Nde1-expressing cells may include type B stem cells. GFAP staining was carried out and the co-expression of GFAP/Nde1 was confirmed (Fig. 3E–G, white arrows). These results suggest that Nde1 may be localized to SVZ-type B stem cells. In contrast, Ndel1 was barely detectable in the SVZ of the lateral ventricle (Fig. 3H, I), which suggested that Ndel1 is probably not expressed in SVZ adult stem cells at a significant level.

### High levels of Nde1 in adult SGZ

**Fig. 4:**
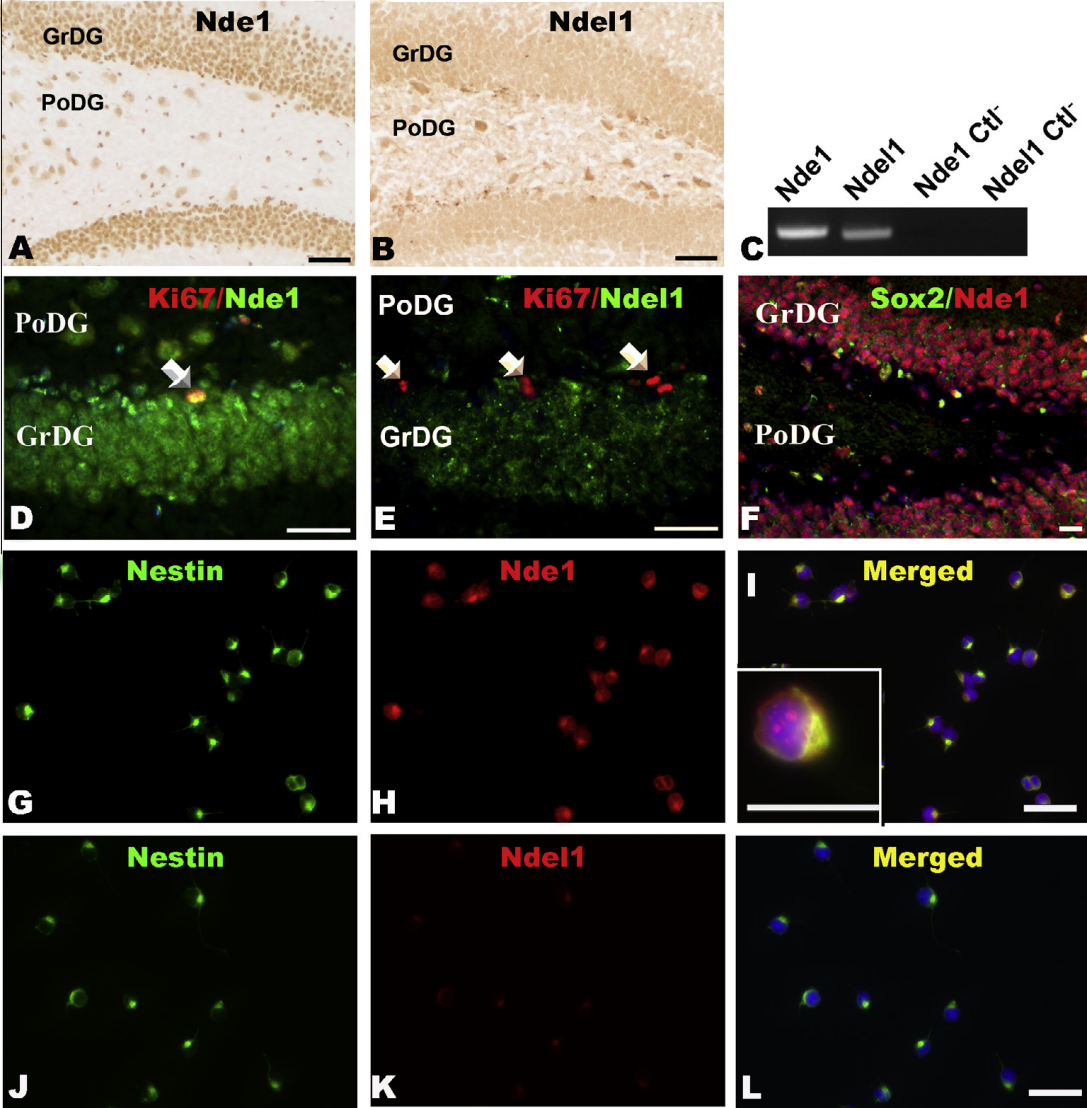
The distribution of Nde1 and Ndel1 in the subgranular zone (SGZ) of the adult hippocampus. (A, B) Immunostaining of the adult mouse hippocampus. Nde1 is highly expressed in both the polymorph layer and granular layer of the dentate gyrus (A); Ndel1 is more highly localized to the somata of some polymorph layer cells, but is also expressed in the granular layer of the dentate gyrus (B). (C) RT-PCR reveals more expression of Nde1 mRNA than Ndel1 mRNA in undifferentiated HCN-A94 cells. (D, E) In the SGZ, Ki67-labeled putative stem cells also express Nde1 (D) but not Ndel1 (E). (F) Double-labeling of Nde1 and Sox2 shows that Sox2-labeled adult stem cells also express Nde1. (G–L) Immunocytochemistry of undifferentiated HCN-A94 cells revealed high expression of Nde1 (G–I) and low expression of Ndel1 (J–L). PoDG, polymorph layer, dentate gyrus; GrDG, granular layer of the dentate gyrus; Ctl−, negative control; Scale bar = 20 μm.

We then examined the expression pattern of Nde1 and Ndel1 in the hippocampal SGZ, another niche where adult stem cells reside (Christian et al., 2010), and found an overlapping, but still distinct pattern (Fig. 4A, B, D–F). Nde1 was expressed in Ki67-positive cells in the SGZ (Fig. 4D). In contrast, there was no or only very weak co-localization of Ndel1 and Ki67 in the same brain region (Fig. 4E). Sox2, a stem cell marker, also was co-expressed in a subset of Nde1-positive cells in the SGZ (Fig. 4F). Data suggest that Nde1 but not Ndel1 is expressed in actively dividing adult stem cells in the SGZ region.

To confirm the potential expression of Nde1 in SGZ adult stem cells, we performed immunocytochemistry and RT-PCR assays in HCN-A94 cells, an adult rat hippocampal neural stem cell line (Gage et al., 1995; Shen et al., 2011). High levels of Nde1 were found in undifferentiated HCN-A94 cells and intriguingly, these Nde1-expressing cells consistently showed co-immunostaining of Nestin, a specific marker for neural stem cells (Fig. 4G–I). In contrast, weak expression of Ndel1 was detected in these cells (Fig. 4J–L). Similar mRNA expression profile was detected by RT-PCR (Fig. 4C). We therefore concluded that Nde1 but not Ndel1 is abundantly expressed in adult hippocampal progenitor (stem) cells.

### Nde1 overexpression decreases astrocytic differentiation of HCN-A94 cells

**Fig. 5:**
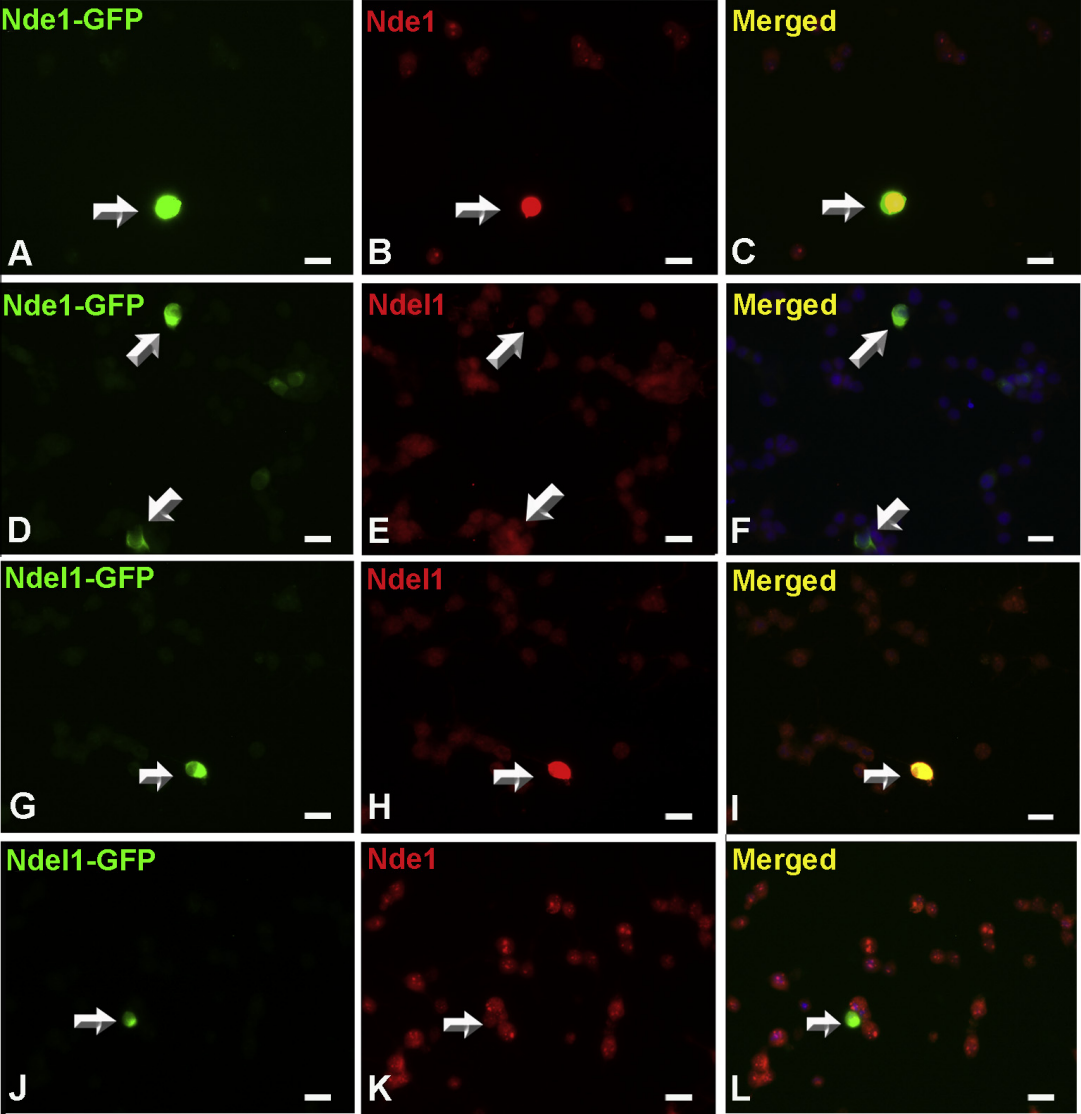
Overexpression of Nde1 in HCN-A94 cells does not affect Ndel1 expression and *vice versa*. HCN-A94 cells were transfected transiently with GFP-expressing plasmids to overexpress Nde1-GFP (A–F) and Ndel1-GFP (G–L). Nde1 or Ndel1 expression was detected in addition to GFP fluorescence. Overexpression of Nde1 did not affect the Ndel1 expression level (D–F). Ndel1 overexpression did not affect the Nde1 protein level (J–L). These experiments further confirm the specificity of both antibodies in immunohistochemistry. Scale bar = 20 μm.

**Fig. 6:**
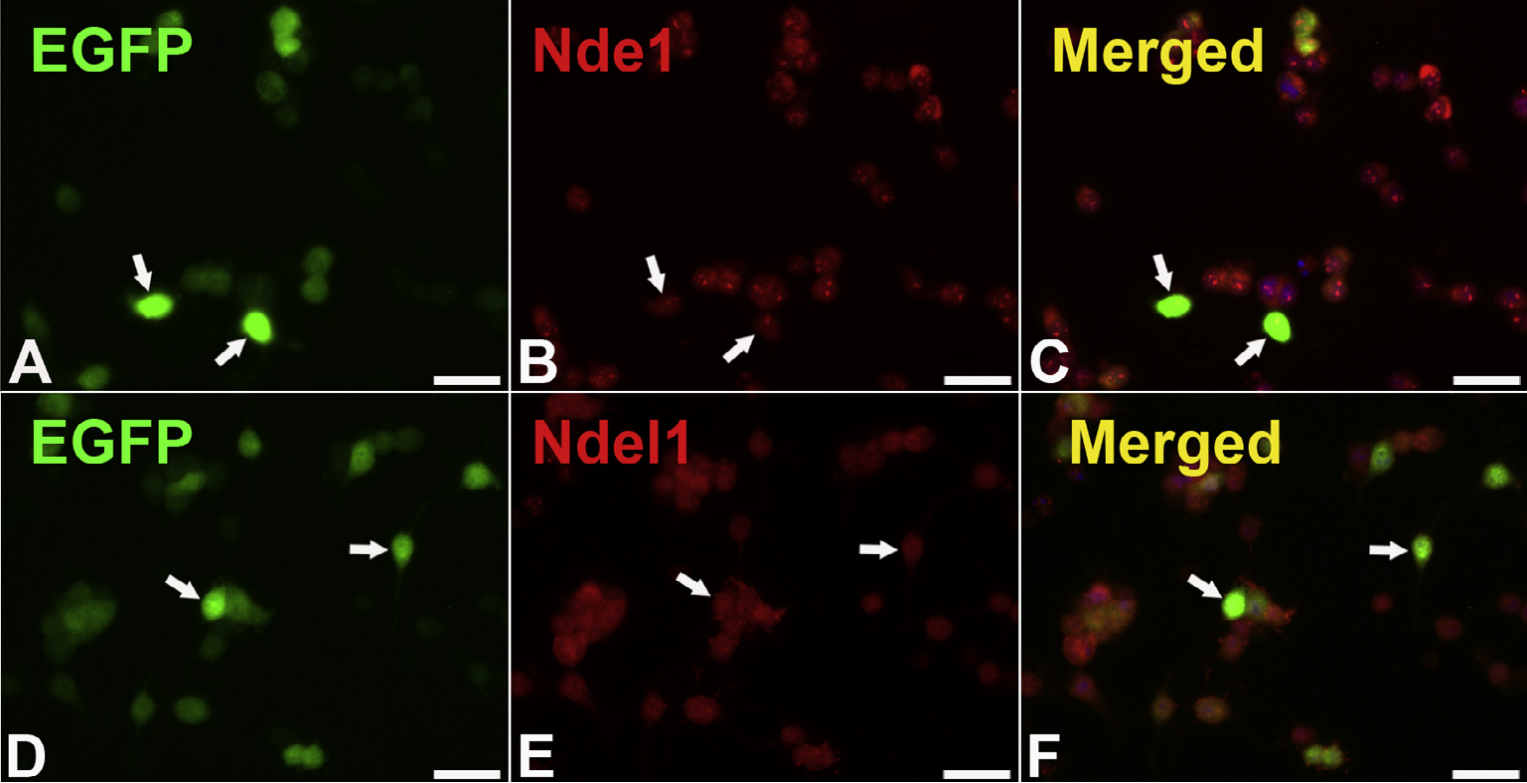
Transfection of HCN-A94 cells with control pCMV-EGFP did not affect the expression of endogenous Nde1 (A–C) or Ndel1 (D–F). Scale bar = 25 μm.

**Fig. 7:**
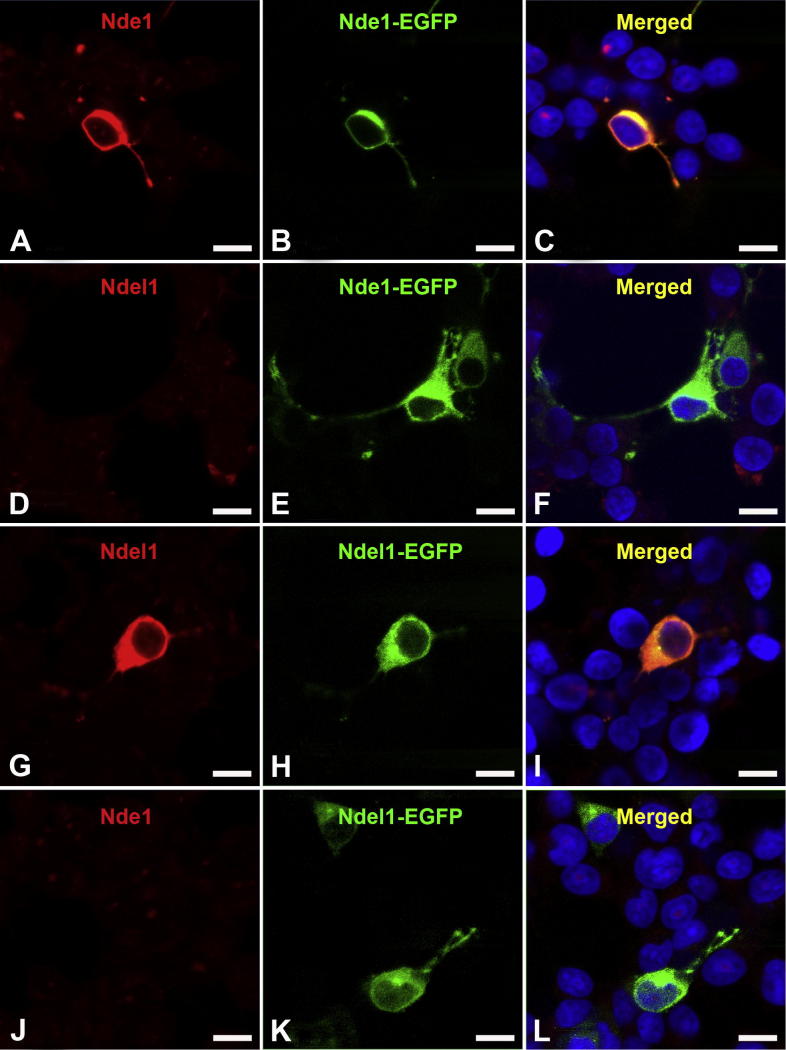
The specificity of antibodies was further demonstrated in overexpression experiments by immunocytochemistry. Cells overexpressing Nde1 (B, C, E, F) can be detected by anti-Nde1 antibody (A) but not by anti-Ndel1 antibody (D). Similarly, cells transfected with pCMV-Ndel1-EGFP (H, I, K, L) can be identified with anti-Ndel1 (G) but not by anti-Nde1 (J). Scale bar = 10 μm.

**Fig. 8:**
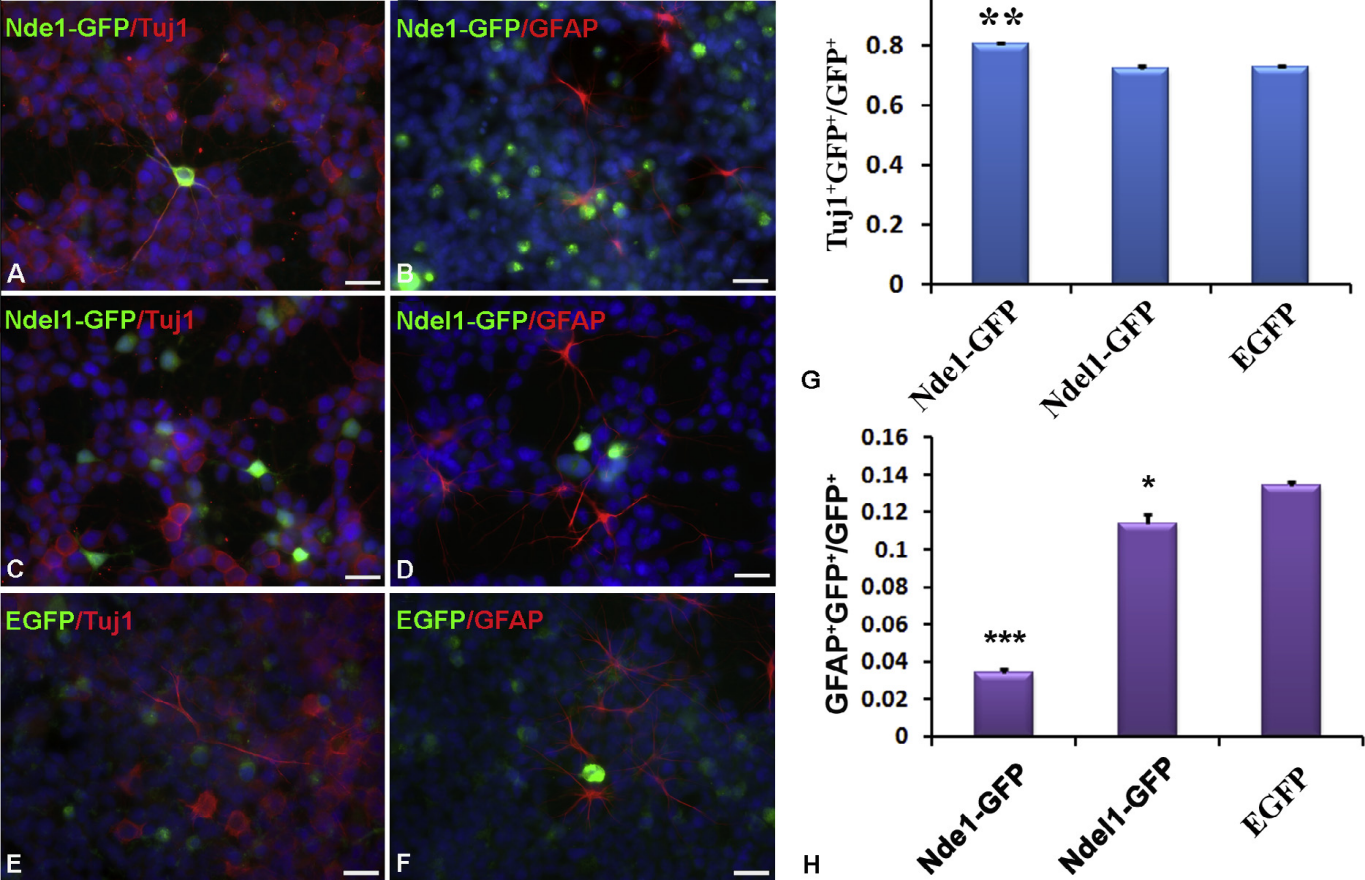
Immunohistochemistry of differentiated HCN-A94 cells. pCMV-Nde1-GFP (A, B), pCMV-Ndel1-GFP (C, D) or negative control pCMV-EGFP (E, F) transfected HCN-A94 cells were differentiated into a mixed population of neurons and glia. The neuronal cells were identified by Tuj1 staining (A, C, E) and the glial cells were identified by GFAP staining (B, D, F). (G, H) Percentages of the neuronal (G) and glial (H) differentiated cells were calculated after plasmid transfection. Only GFP-expressing cells (total number: *n* > 15,000) were counted. ^∗^*P* < 0.05; ^∗∗^*P* < 0.005; ^∗∗∗^*P* < 0.001; Scale bar = 20 μm.

The different expression patterns of Nde1 and Ndel1 in the adult mouse hippocampus suggest that Nde1 and Ndel1 may have different functions during adult neurogenesis. In order to examine the precise functions of Nde1 and Ndel1 in adult hippocampal stem cells, and to determine how CNV of Nde1 and Ndel1 in the human population may underlie abnormal brain development, we have generated specific plasmids to over-express rat full length *Nde1* or *Ndel1* and performed the transfection experiments. Using the pCMV-EGFP, pCMV-Nde1-EGFP or pCMV-Ndel1-EGFP expression constructs described earlier, each gene was transiently over-expressed independently in undifferentiated HCN-A94 cells and protein up-regulation was detected by immunocytochemistry (Fig. 5). Nde1 overexpression increased Nde1 protein levels as expected (Fig. 5A–C) but did not affect the expression of Ndel1 (Fig. 5D–F). The same pattern was also demonstrated in Ndel1-overexpressing cells (Fig. 5G–L). None of the empty EGFP-vector transfected cells showed a significant increase in Nde1 or Ndel1 proteins (Fig. 6). Further confocal analyses of differentiated HCN-A94 cells have also demonstrated the specificity of the antibodies we have used (Fig. 7). To determine the roles of Nde1 and Ndel1 during adult stem cell differentiation, the transfected HCN cells were differentiated for 6 days in serum-containing medium as described before, and then immunostained with anti β-tubulin III (Tuj1) (Fig. 8A, C, E) and anti-GFAP (Fig. 8B, D, F). The percentage of Tuj1/GFP-positive and GFAP/GFP-positive was measured in transfected cells. Compared to EGFP vector-only transfected cells, Nde1-overexpression significantly decreased the number of differentiated astrocytes (*P* < 0.001), whereas the number of differentiated neurons was increased slightly (Fig. 8G, H). A smaller, but still significant, decrease in the number of astrocyte was observed also in Ndel1-overexpressing cells (*P* < 0.05). These results strongly suggest that the Nde1 and Ndel1 protein levels modulate the neuron/astrocyte differentiation decision in differentiating HCN-A94 cells and Nde1, which shows high levels of expression in hippocampal stem cells, has a more predominant effect than Ndel1. It should be noted that our immunohistochemistry indicates that the level of fusion protein produced from the plasmids is far greater than that normally found in un-transfected HCN-A94 cells, possibly by an order of magnitude. It is prudent therefore to be cautious about extrapolating these supranormal dosages to the mechanism of human neurological disease.

### Subcellular distribution of Nde1 and Ndel1 in cultured cells

**Fig. 9:**
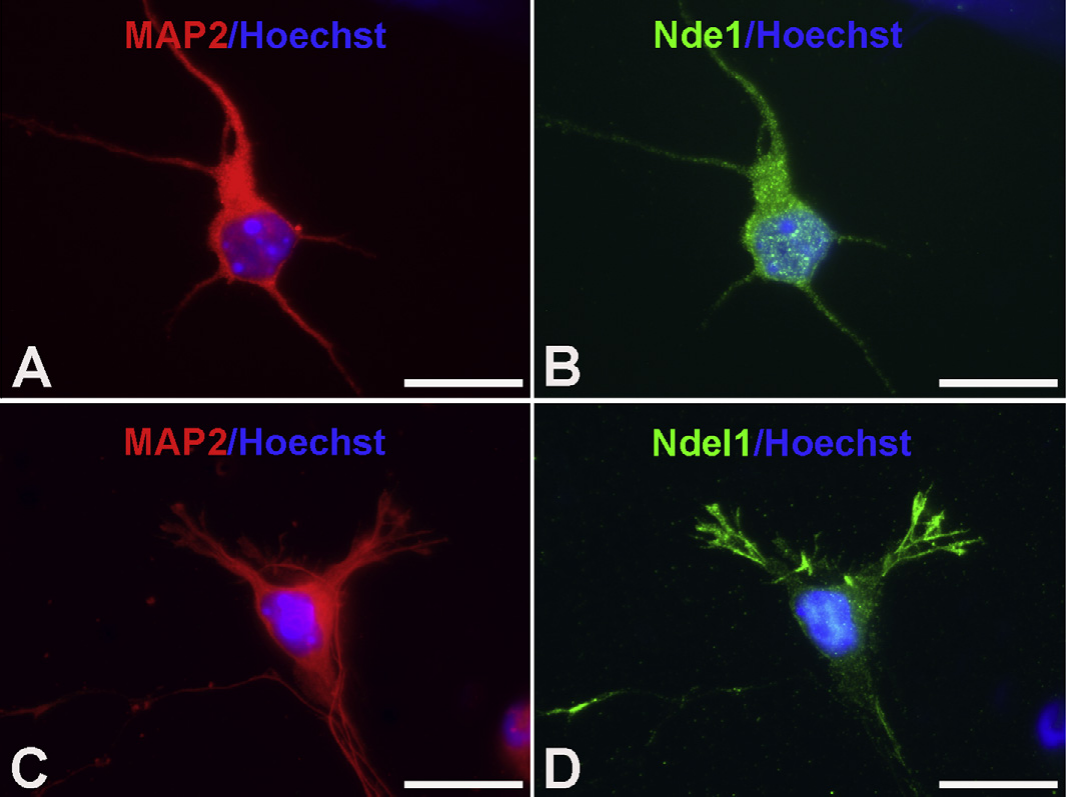
Expression of Nde1 and Ndel1 in primary cultured neurons. (A–D) Endogenous Nde1 and Ndel1 were detectable in primary cultured neurons (MAP2-positive, red). Nde1 was extensively located in the cytoplasm, nucleus and proximal neurites (A, B). However, Ndel1 was expressed in the cytoplasm and nucleus and distal neurites, including growth cones (C, D). (For interpretation of the references to color in this figure legend, the reader is referred to the web version of this article.)

**Fig. 10:**
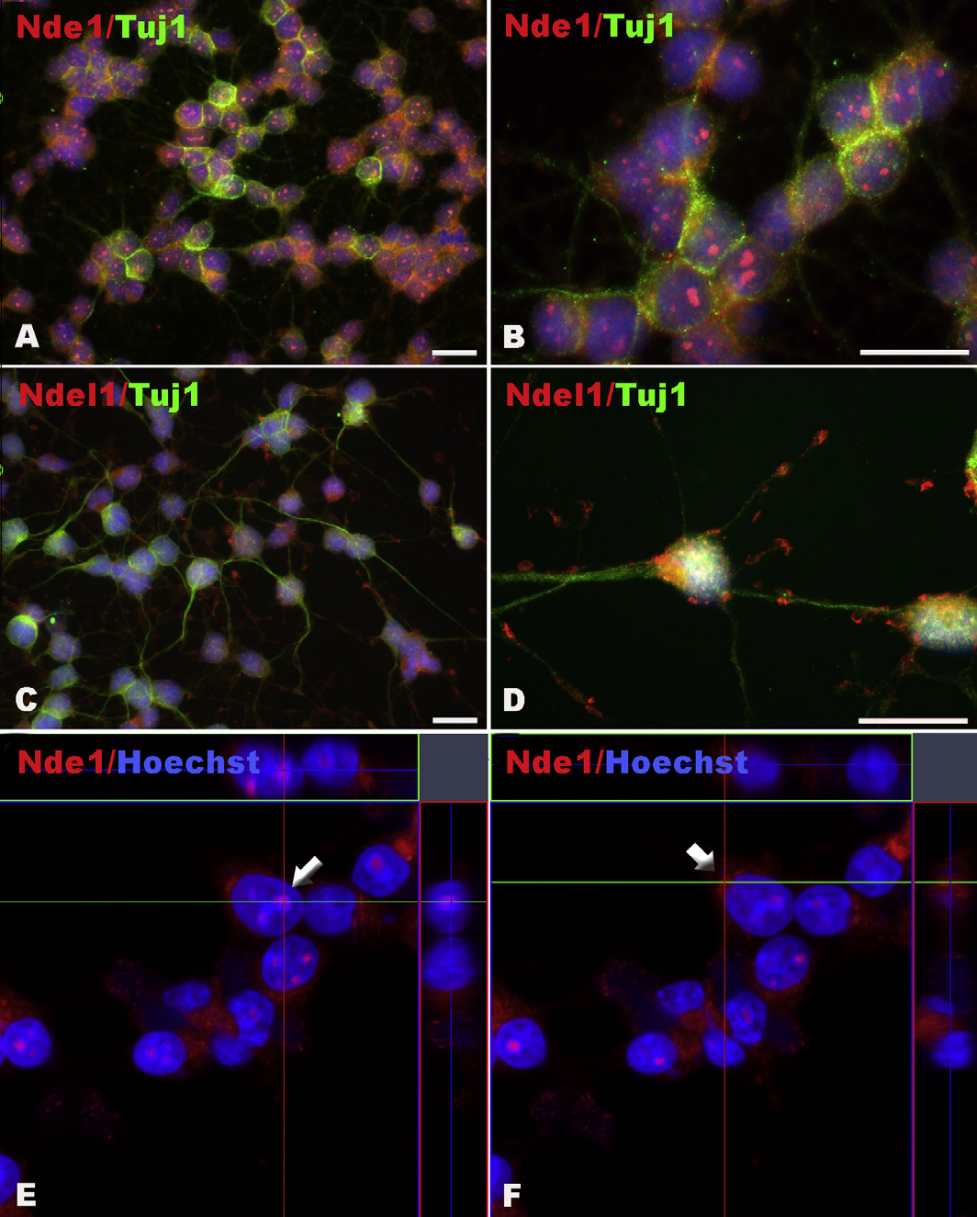
The distribution profiles of Nde1 and Ndel1 in neuronal-differentiated HCN-A94 cells. (A–F) Nde1 (A, C, E) and Ndel1 (B, D, F) are both highly expressed in neuronally-committed HCN-A94 cells after differentiation for 4 days. (D) Ndel1 also was detectable in nuclei but more obviously expressed in the growth cones. Confocal microscopy shows the localization of Nde1 within nucleoli (E) and the perinuclear region (F). Scale bar = 20 μm.

We next examined the detailed subcellular localization of Nde1 and Ndel1 in cultured cells. In primary cultured neurons, we observed similar subcellular localization of Nde1 (Figs. 9A, B and 10E, F) as in previous reports (Bradshaw et al., 2009, 2011). While Nde1 was extensively located in the cytoplasm and nuclear or perinuclear areas, Ndel1 was present significantly in axonal and dendritic extensions as well as growth cones (Fig. 9C, D). Similar distribution patterns were also found in neuronal-differentiated HCN-A94 cells (Fig. 10A–F).

**Fig. 11:**
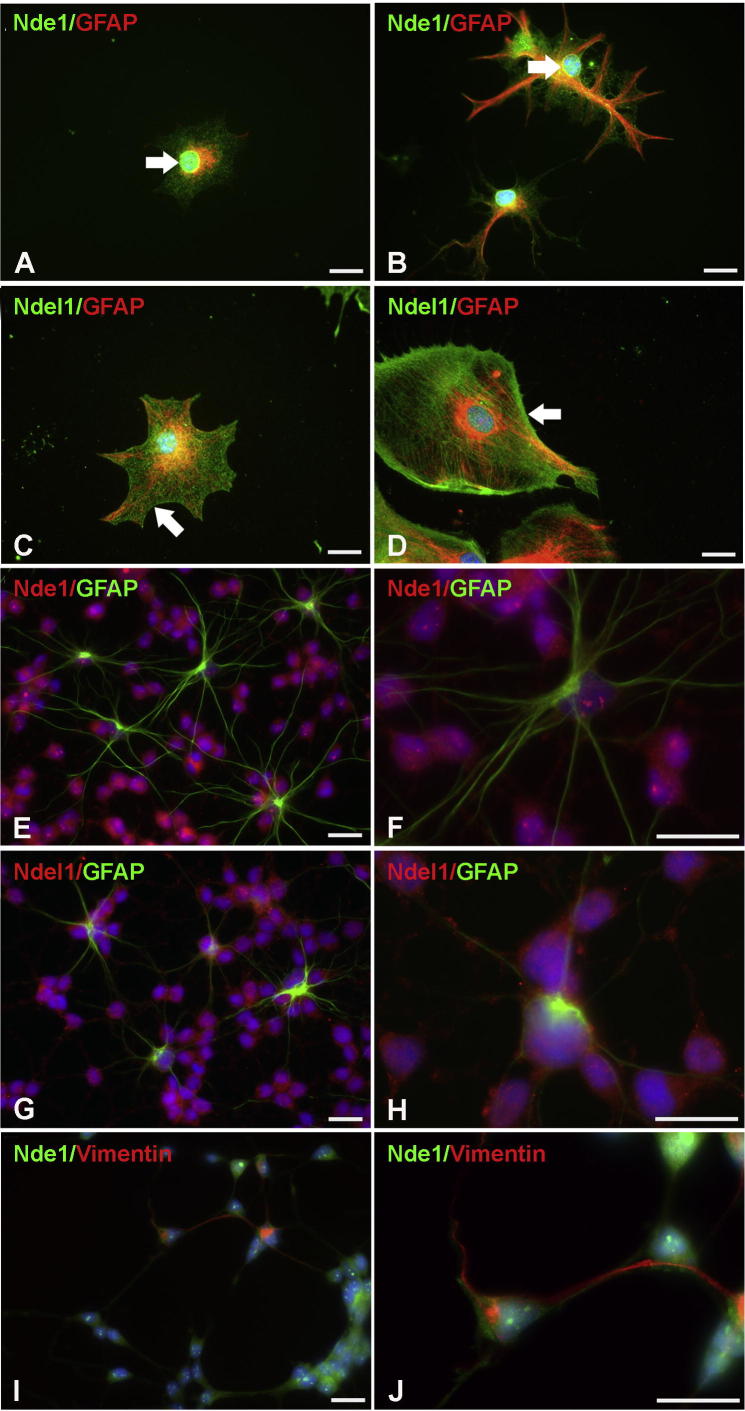
The astrocytic expression of Nde1 and Ndel1. (A, B) Immunocytochemistry on primary cultured cortical astrocytes showed the expression of Nde1 (A, B) and Ndel1 (C, D). Nde1 was located strongly in the nuclei and also in the cytoplasm (A, B). Ndel1 was expressed extensively in nuclei, cytoplasm and also cellular processes (C, D). (E–H) Astrocytes differentiated from HCN-A94 cells express Nde1 and Ndel1. (I, J) Nde1 protein is detectable in vimentin-positive immature astrocytes. Scale bar = 20 μm.

Astrocytes have critical roles in brain function and tissue homeostasis and abnormalities in their function are linked to disorders including schizophrenia and epilepsy. Interestingly, subcellular localization of Nde1 and Ndel1 in astrocytes has not been reported before. In light of the evidence presented above that Nde1 dosage may affect astrocytic differentiation, we performed this examination by immunocytochemistry and detected the expression of Nde1 and Ndel1 in primary cultured astrocytes generated from the cortex of E18.5 mice (Fig. 11A–D). Nde1 and Ndel1 were found to have different subcellular localizations in astrocytes, similar to those observed in primary cultured neurons. While Nde1 was highly expressed in nuclei and also the perinuclear region (Fig. 11A, B, white arrows), Ndel1 was mostly distributed in the cytoplasm, especially in filopodial processes of astrocytes (Fig. 11C, D, white arrows). These localization patterns broadly recapitulated their subcellular localization in neurons. Meanwhile, Nde1 and Ndel1 were also found in astrocytic HCN-A94 cells after differentiation (Fig. 11E–J). It was demonstrated that Nde1 showed cytoplasmic localization primarily in vimentin-positive cells (Fig. 11I, J), a cell marker for immature astrocytes. Nde1 protein level and localization therefore may change during glial differentiation. Our results suggest that both Nde1 and Ndel1 exist in multiple astrocytic morphological subtypes, but their subcellular distributions do not overlap extensively.

### Neuronal and astrocytic distributions of NDE1 in the adult human cortex

**Fig. 12:**
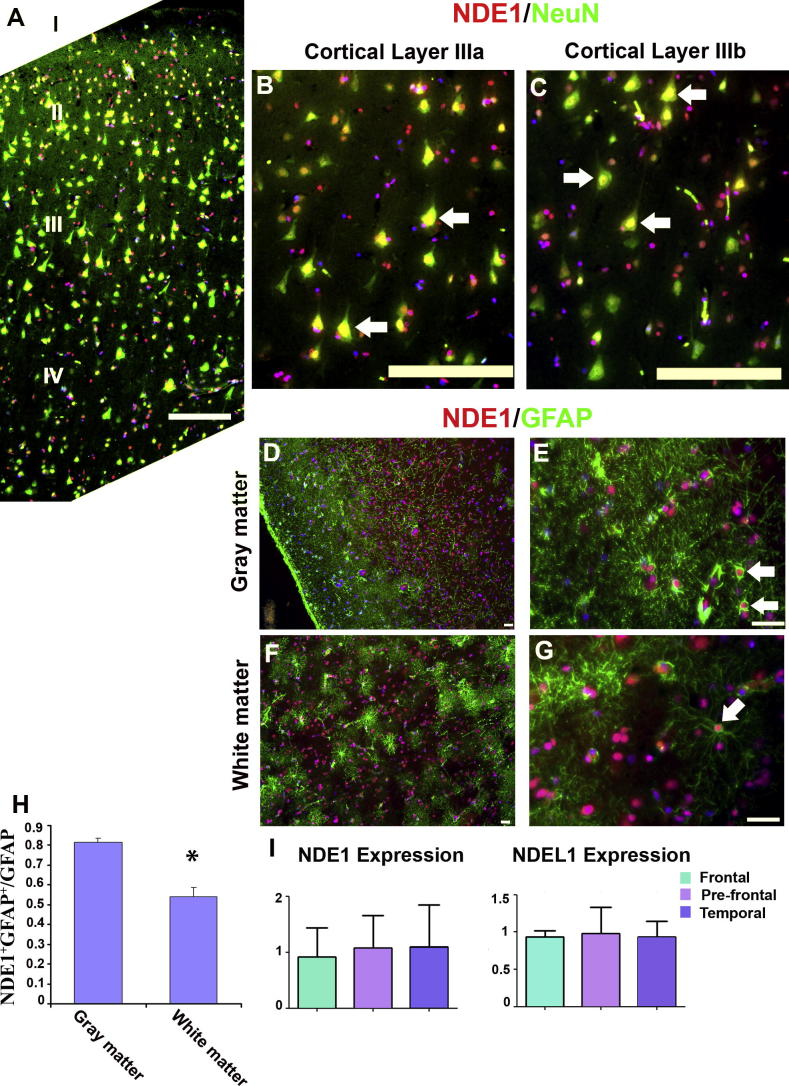
NDE1 distribution profiles in the human cortex. (A–C) Double immunolabeling of human cortical slices shows that NDE1 is distributed extensively in NeuN-positive neurons in all layers of the cortex. Typical double-labeled neurons are indicated with white arrows. (D–G) NDE1 is also distributed in cortical astrocytes. In gray matter, subsets of GFAP-expressing astrocytes are labeled with NDE1 (E, white arrows). (F, G) GFAP and NDE1 double-labeled cells also were detectable in cortical white matter. (H) The proportion of NDE1-expressing astrocytes was significantly greater in gray than in white matter of the human cortex (10 samples from five donors; Mann–Whitney *U* test (*P* < 0.02)). (I) qPCR experiments also revealed the mRNA expression of NDE1 and NDEL1 in the three different cortical regions (frontal, prefrontal and temporal lobe) with no significant variations (*n* = 3). Scale bar = 20 μm.

Surprisingly, there is almost no histological description of the localization of NDE1 or NDEL1 in the adult human brain. To address this question, immunohistochemical staining was performed on human cortical sections. Although the Ndel1 antibody did not produce any specific staining in these sections, we successfully detected NDE1 in the prefrontal, frontal and temporal lobes of the adult human cortex. NDE1 protein was distributed in both cortical neurons (Fig. 12A–C) and astrocytes (Fig. 12D–G). In all cortical layers, NDE1 was distributed broadly in the majority of NeuN-labeled mature neurons, while some GFAP-expressing astrocytes expressed NDE1 (Fig. 12E, G, white arrows). Interestingly, we found that gray matter of human cortex contained more NDE1-positive astrocytes than white matter (Fig. 12H). Because most gray matter astrocytes are protoplasmic while most of the white matter astrocytes are of the fibrous type, our results suggest that NDE1 is heterogeneously expressed in different astrocyte subtypes. This heterogeneous NDE1 localization was consistently found in the cortical regions of all four donors.

qPCR experiments were then carried out to compare the mRNA expression abundance of NDE1 and NDEL1 in different human cortical regions. The assay included samples from the prefrontal, frontal and temporal cortices. After normalization against controls, no significant variation was demonstrated among these regions for either NDE1 or NDEL1 (Fig. 11I), suggesting that both NDE1 and NDEL1 are widely and homogenously expressed in the human cortex. Our results convincingly show that NDE1 protein is distributed widely in the human cortex, as expected, but is localized heterogeneously in astrocytes in gray and white matters.

## Discussion

Neurological diseases such as schizophrenia and epilepsy are highly complex disorders. Their etiology and its relationship with therapeutics used for treatment are still poorly understood. NDE1 in particular is genetically associated with both diseases. In this study, Nde1 and Ndel1 protein distribution and function have been compared in both neurons and astrocytes, to address some outstanding questions about their cellular and subcellular localization in the mouse and human CNS, and to investigate possible roles in modulating the differentiation of neurons and glia from neural stem cells.

Nde1 and Ndel1 are essential for neuronal development and are important cell cycle regulators (Mori et al., 2007; Kim et al., 2011). Most studies have focused on their functions during brain development, including the proliferation of neural precursor cells and neuronal migration (Bengzon et al., 1997; Sasaki et al., 2005; Taya et al., 2007; Kim et al., 2011). Nde1 deletion leads to cortical developmental defects (Feng and Walsh, 2004; Pawlisz et al., 2008), and these abnormalities are mostly correlated with defective embryonic neurogenesis. Here, we have shown that both Nde1 and Ndel1 are expressed broadly in adult mouse and human brains, including the majority of cortical neurons. This expression pattern of both proteins suggests that they have important functions in mature neurons. Additionally, although Nde1 regulates radial glial functions during development (Bengzon et al., 1997) and its function is pivotal for human glioma formation (Suzuki et al., 2007), the astrocytic distribution of Nde1 and Ndel1 has not been investigated extensively. We have demonstrated that Nde1 and Ndel1 are expressed in multiple subtypes of adult astrocytes with different morphological characteristics both in mice and humans. Our *in vitro* studies further showed that Nde1 and Ndel1 have similar subcellular localization in astrocytes which is comparable to their distribution pattern in neurons.

Based on the multi-compartmental existence of Nde1 and Ndel1 in astrocytes, together with their important roles during cell division and spindle orientation (Feng and Walsh, 2004), we speculate that they may be critical for brain homeostasis and therefore contribute to a range of disease states. Interestingly, NDE1 and NDEL1 have been found in gliomas and their expression is highly associated with the activity of glioma cell migration and proliferation (Suzuki et al., 2007). We also found a heterogeneous expression of NDE1 in the gray matter and white matter of the human brain, and also in different sub-layers of the human cortex. These results suggest that NDE1 is expressed selectively in certain populations of adult cortical astrocytes, although the properties of these astrocytic subtypes require further study.

Intriguingly, *NDE1* CNV has been classified as a potential risk factor for a series of neuronal disorders including schizophrenia and epilepsy. Ndel1 regulates aspects of embryonic neuronal migration and neurogenesis and cooperates with its binding partner DISC1 in regulating adult neurogenesis. Knockdown of Ndel1 in the SGZ leads to abnormal soma size and migration defects (Parent et al., 1997). In contrast, the function of Nde1 in adult neurogenesis had not been studied. We found that Nde1 rather than Ndel1 was highly expressed in GFAP-positive SVZ cells with radial-like processes. This suggests Nde1 is present in SVZ-type B stem cells. In the SGZ, Nde1, but not Ndel1, was co-localized with Ki67 in actively dividing neural stem cells. These data suggest that Nde1 is more likely than Ndel1 to be involved in adult neural stem cell function. HCN-A94 cells can be differentiated into a mixed cell population containing neurons and astrocytes. In this study, Nde1 overexpression resulted in the decreased ability of HCN-A94 cells to differentiate into astrocytes compared to the control. This suggests that Nde1 and Ndel1 may participate in cell fate determination during adult hippocampal stem cell differentiation and potentially links with observations of CNV of the genes underlying human disease. Our findings are supported by previous experimental data suggesting that Nde1 has an important regulatory function in the dividing cells. This is further evidenced by the small cerebral cortex phenotype observed in *Nde1^−/−^* mice, which was apparently caused by mitotic spindle defects (Feng and Walsh, 2004). Our results suggest that it might be important in future to investigate whether Nde1-overexpressing neural stem cells have mitotic spindle defects and how this alteration modulates their mitotic delay or cell fate specification.

## Conclusions

We have provided the most comprehensive documentation of the expression patterns of Nde1 and Ndel1 in cultured cells as well as in mouse and human brains, and also highlight that dosage effects of these two proteins might contribute to some cases of mental disorder.

## Conflict of interest statement

The authors declare that they have no competing interests.

## Figures and Tables

**Table 1:** The distribution of Nde1 and Ndel1 in the mouse CNS

Observed regions	Intensity	
*Main olfactory bulb*		
EPI (external plexiform layer of the olfactory bulb)	+	+
GA (granular layer)	+	−−
Gl (glomerular layer)	++	+
IPI (internal plexiform layer of the olfactory bulb)	+	+++
Mi (mitral cell layer)	++	+

*Accessory olfactory bulb*		
EPIA (external plexiform layer of the accessory olfactory bulb)	++	−−
MiA (mitral cell layer)	++	+
AOB (Accessory olfactory bulb)	++	+
AOD (anterior olfactory nu, dorsal)	++	+
AOE (anterior olfactory nu, external)	++	−−
AOL (anterior olfactory nu, lateral)	++	+
AOM (anterior olfactory nu, med)	++	+
AOP (anterior olfactory nu, posterior)	++	+
Calleja (island of Calleja)	++	++
DTT (dorsal tenia tecta)	++	++
VTT (ventral tenia tecta)	++	++

*Cerebral cortex*		
AI (agranular insular cortex)	++	++
Au1 (primary auditory cortex)	++	++
AuD (auditory cortex, dorsal part)	++	++
AuV (auditory cortex, ventral part)	++	++
Cg1 (cingulate cortex, area2)	++	+++
Cg2 (cingulate cortex, area2)	++	+++
DI (dysgranular insular cortex)	++	++
DIEnt (dorsal intermediate entorhinal cortex)	++	++
DLO (dorsolateral orbital cortex)	++	++
DP (dorsal peduncular cortex)	++	+++
Ect (ectorhinal cortex)	++	+++
Fr3 (frontal cortex, area3)	++	++
FrA (frontal association cortex)	++	++
GI (granular insular cortex)	++	++
IL (infralimbic cortex)	++	++
LEnt (lateral entorhinal cortex)	+++	+++
LO (lateral orbital cortex)	++	++
LPtA (lateral parietal association cortex)	++	++
M1(primary motor cortex)	++	+++
M2 (secondary motor cortex)	++	+++
MEnt (medial entorhinal cortex)	+++	+++
MO (medial orbital cortex)	++	+++
MPtA (medial parietal association cortex)	++	+++
Pir (piriform cortex)	+++	+++
PPtA (posterior parietal assoc area)	++	+++
PRh (perihinal cortex)	++	+++
PrL (prelimbic cortex)	++	+++
RSA (retrosplenial agranular cortex)	++	+++
RSG (retrosplenial granular cortex)	++	+++
S1 (somatosensory cortex)	++	++
S1BF (somatosensory1, barrel field)	++	++
S1DZ (primary somatosensory cortex, dysgranular zone)	++	++
S1DZO(primary somatosensory cortex, oral dysgranular zone)	++	++
S1HL (primary somatosensory cortex, oral dysgranular zone)	++	++
S1J (somatosensory1, jaw reg)	++	++
S1Tr (somatosensory1, trunk reg)	++	++
S1ULp (primary somatosensory cortex, upper lip region)	++	++
S2 (secondary somatosensory cortex)	++	++
TeA (temporal cortex, association a)	++	++
V1 (primary visual cortex)	++	++
V1B (primary visual cortex, binoc)	++	++
V1M (primary visual cortex, binoc)	++	++
V2L (secondary visual cortex)	++	++
V2ML (visual cortex2, mediolat)	++	++
V2MM (visual cortex 2, meduomed)	++	+++
VO (ventral orbital cortex)	++	++

*Hippocampus*		
CA1, pyramidal cell layer	++	++
CA2/CA3, pyramidal cell layer	++	+++
FC (fasciola cinereum)	++	++
GrDG (granular layer, dentate gyrus	++	+
LMol (lacunosum molecular layer, hippocampus)	+	+
MoDG (molecular layer of the dentate gyrus)	+	+
Or (oriens layer, hippocampus)	+	+
PoDG (polymorph layer, dentate gyrus	++	+++
Rad (stratum radiatium, hippocampus)	+	+
S (subiculum)	++	+++
SHi (septohippocampal nu)	++	+++
SLu (stratum lucidum, hippocampus)	+	+

*Basal Ganglia*		
Cl (claustrum)	−−	−−
GP (globus pallidus)	++	−−
Striatum/CPU (caudate putamen)	+++	+++
SN (Substantia nigra, SNC/SNR)	+	+
Acbc (accumbens nucleus)	++	+

*Septal region*		
LS (lateral septal nu, LSD/LSV)	++	++
SFi (septofimbrial)	++	+
SHi (septohippocampal nu)	+	+
MS (medial septal nu)	++	++
DB (nucleus of diagonal band, HDB/VDB)	++	+
BST (Bed nucleus of the stria terminalis)	++	+
HDB (nu horiz limb diagonal band)	++	+++

*Amygdala*		
Co (anterior cortical amygdaloid nu, Aco/PMCo)	++	++
AHi (amygdalohipp area, AHiAL/AHiPM)	++	++
AStr (amygdalostriation transition area)	++	++
BL (basolat amygdaloid nu, BLA/BLV/BLP)	++	++
BM (basomed amygdala, BMA/BMP)	++	++
Ce (cent amygdaloid nu, CeC/CeL/CeMPV)	++	++
I (intercalated nuclei amygdala)	++	++
La (lat amygdaloid nu, LaDL/LaVL/LaVM)	++	++
MeP (med amygdaloid nu, MePD/MePV)	++	++

*Dorsal Thalamus*		
VL (ventrolateral thalamic nu)	+	+
PV (paraventricular thalamic nu)	+	++
MD (mediodorsal thalamic nu, MDC/MDL/MDM)	+	++
LD (laterodorsal thalamic nu, LDDM/LDVL)	+	+
VPM (ventral posteromed thalamic nu)	+	++
VPL (ventral posterolat. Thalamic nu)	+	++
CL (central lateral thalamic nu)	+	+
SM (nucleus stria medullaris)	+	++
IMD (intermediodorsal thalamic nu)	+	++
Re (reuniens thalamic nu)	+	++
VM (ventromed thalamic nu)	+	−−
CM (central medial thalamic nu)	+	++
SCO (subcomissural organ)	+	−−
Dk (nu of Darkschewitsch)	+	++
EW (Edinger–Westphal nu)	+	++
MPO (med proptic nu, MPOM/MPOC/MPOL)	+	++

*Epithalamus and hypothalamus*		
STh (subthalamic nu)	+	+
MHb (med habenular nu)	+	−−
LHb (lateral habenular nu)	+	−−
AHP (anterior hypothal area, posterior)	++	+
Arc (arcuate hypothal nu, ArcD/ArcL)	+	+
DM (dorsomedial hypothalamic nu)	+	+
LH (lateral hypothal area)	+	+
MM (mammillary nuclei, SuMM/MMn/LM/SuML/LM)	++	++
PaDC (paravent hypothal dorsal cap)	++	++
PaLM (paravent hypothal lat magnocell)	++	++
PaMM (paravent hypothal med magnocell)	++	++
PaMP (paravent hypothal med parvicell)	++	++
RCh (retrochiasmatic area)	+	+
TC (tuber cinereum area)	+	+
VMH (ventromed hypothal nu VMHDM/VMHC/VMHVL)	+	++
VTA (ventral tegmental area)	+	+
ZI (zona incerta, ZID/ZIV)	−−	−−
		
*Mesencephalon and rhombencephalon*		
Superior colliculus		
DpG (deep gray layer, sup colliculus)	+	++
InG (intermed gray layer sup colliculus)	+	++
InWh (intermed white layer sup colliculus)	+	++
OP (optic nerve layer sup colliculus)	+	+
SuG (superficial gray, sup colliculus)	+	+
Zo (zonal layer superior colliculus)	+	+

*Inferior colliculus*		
ECIC (external cortex, infer colliculus)	−−	+
CIC (central nu inferior colliculus)	−−	−−
DCIC (dorsal cortex, inferior colliculus)	−−	−−

*Precerebellar nu and red nu*		
R (red nucleus, RPC/RMC)	++	++
PnV (pontine reticular nu)	+	+
Gi (gigantocellular reticular nu)	+	+
LPGi (lateral paragigantocellular nu)	+	+
DPGi (dorsal paragigantocellular nucleus)	+	+
Pn (pontine nuclei)	+	++
PMn (paramedian reticular nu)	++	++
LRt (lateral reticular nu)	+	++
Irt (intermediate reticular nu)	+	++
LSO (lateral superior olive)	++	+
LVPO (lateral ventral periolivary nu)	++	+
DPO (dorsal periolivary region)	++	+
MVPO (mediovent periolivary nu)	++	+
SPO (superior paraolivary nu)	++	+
RPO (rostral periolivary region)	++	+

*Periaqueductal gray*		
PAG (periaqueductal gray)	++	+
DLPAG (dorsal lat periaqueductal gray)	++	+
DMPAG (dorsomed periaqueductal gray)	++	+
LPAG (lateral periaqueductal gray)	++	+
VLPAG (ventrolat periaqueductal gray)	++	+

*Oromotor nuclei*		
Mo5 (motor nucleus of the trigeminal nerve)	+	+
Seven (facial nu)	+	+
Twelve (hypoglossal nu)	+	++

*Locus coeruleus*		
LC (locus coeruleus)	++	++

*Raphe nucleus*		
DR (dorsal raphe nucleus, DRC/DRD/DRI/DRV)	+	+
Raphe nucleus pallidus (RPa)	+	+
Raphe nucleus obscurus (Rob)	+	+
ROb (raphe obscurus nu)	+	++
RPa (raphe pallidus nu)	+	++
DRC (dorsal raphe nu, caudal)	+	+
DRI (dorsal raphe nu, inferior)	+	+
RMg (raphe magnus nu)	+	+

*Parabrachia nucleus*		
LPB (lateral parabrachial nu, LPBC/LPBD/LPBE/LPBV)	+	+
MPB (medial parabrachial nu)	+	+

*Tegmental Nucleus*		
LDTg (laterodorsal tegmental nu)	+	+
PDTg (posterdorsal tegmental nu)	++	++

*Vestibular nucleus*		
SpVe (spinal vestibular nucleus)	+	++
Mve (medial vestibular nu)	+	++
LVe (lateral vestibular nu)	+	++

*Cochlear nucleus*		
SGl (superficial glial layer, cochlear nu)	+	++
AVC (anteroventral cochlear nu)	+	++

*Others*		
IP (Interpeduncular nucleus)	+	+
Six abducens nu	+	+
Ten dorsal motor nu vagus nu	++	++
Sol (solitary tract nu)	++	+
Me5 (mesencephalic trigeminal nucleus)	+	++

*Cerebellum*		
Molecular layer	−−	−−
Purkinje cell layer	++	++
Granular layer	−−	−−

*Note:* Intensity of immunohistochemical staining: + ++, high; + +, moderate; +, low; −, negative. The anatomical nomenclature of Paxinos and Watson (1986) is adopted in the most regions of the CNS.
